# O-Antigen Protects Gram-Negative Bacteria from Histone Killing

**DOI:** 10.1371/journal.pone.0071097

**Published:** 2013-08-08

**Authors:** Catherine Chaput, Eileen Spindler, Ryan T. Gill, Arturo Zychlinsky

**Affiliations:** 1 Department of Cellular Microbiology, Max-Planck Institute for Infection Biology, Berlin, Germany; 2 Department of Chemical and Biological Engineering, University of Colorado, Boulder, Colorado, United States of America; The Scripps Research Institute and Sorrento Therapeutics, Inc., United States of America

## Abstract

Beyond their traditional role of wrapping DNA, histones display antibacterial activity to Gram-negative and -positive bacteria. To identify bacterial components that allow survival to a histone challenge, we selected resistant bacteria from homologous *Escherichia coli* libraries that harbor plasmids carrying pieces of the chromosome in different sizes. We identified genes required for exopolysaccharide production and for the synthesis of the polysaccharide domain of the lipopolysaccharide, called O-antigen. Indeed, O-antigen and exopolysaccharide conferred further resistance to histones. Notably, O-antigen also conferred resistance to histones in the pathogens *Shigella flexneri* and *Klebsiella pneumoniae*.

## Introduction

Natural antimicrobial peptides (AMPs) are thought to regulate both the symbiotic flora on the mucosa and immune defense, for example through the recruitment of inflammatory cells [1]–[3]. AMPs are stored in lysosomes and granules of macrophages and neutrophils respectively. Defensins, for example, are often secreted by epithelial cells. Interestingly, histones are one of the most potent antimicrobials isolated from fish and toads [4]–[11]. In eukaryotes DNA is wrapped around a core complex of histones, H2A, H2B, H3 and H4 forming a nucleosome. The linker histone H1, compacts nucleosome allowing the cell to contain and organize its DNA. Histones are also found in the cytoplasm, for example in the granules of human neutrophils [10], [12]. Extracellularly, histones fragments were identified as antimicrobials in the mucosal surfaces of fish, toad, pig, cow and human stomachs [13]. In human and mice, histones are released into the bloodstream during severe sepsis [14], [15].

Microbes have different susceptibilities to histones, but the characteristics that determine this phenotype are not known [5]. To identify genes that confer resistance to histones, we selected histone resistant clones from libraries that over-express random genomic fragments in *Escherichia coli.* We validated our results by the controlled expression of selected genes, or groups of genes, and biochemical analysis. We show that production of smooth lipopolysaccharide (LPS, O-antigen^+^) and exopolysaccharide restricts susceptibility to histones in *E. coli*. Exopolysaccharides are polymers of sugars secreted by bacteria that build a capsule or pseudo-capsule. LPS is an outer membrane component of Gram-negative bacteria composed of 3 domains: a lipid A, a core and an O-antigen. The O-antigen is a complex polysaccharide exposed at the bacterial surface and varies from strain to strain. We showed that the protective function of the O-antigen towards histones is operative in the Gram-negative pathogenic bacteria, *Shigella flexneri* and *Klebsiella pneumoniae,* which cause dysentery and pneumonia respectively.

## Materials and Methods

### Bacterial Strains and Growth Conditions

We used the *E. coli* strains MACH1-T1® (referred to as MACH1) and TOP10 (from Invitrogen), *S. flexneri* strains M90T and the isogenic *waaL* and *rfbA* mutants (Manuscript in preparation: Geldmacher *et al.*), *K. pneumoniae* strains, wild type 52145, the unencapsulated (52K10) or O-antigen- (52O21) mutants, were kindly obtained from Regis Tournebize and Jose A. Bengoechea [16]. Bacteria were grown in Lysogeny Broth (LB) media, except for *S. flexneri,* which was grown in TSB. Medium was supplemented, when necessary, with kanamycin (50 µg/ml, Km), chloramphenicol (50 µg/ml, Cm) and/or L-arabinose. Histones from calf thymus (Calbiochem, H9250) were used as provided by the manufacturer or after re-purification by chromatography (Histone purification kit, Active Motif).

### Selection Procedure

The *E. coli* libraries consisted of MACH1 pSMART-LCKm carrying random fragments of 1, 2, 4 and 8 kb of *E. coli* genome as previously described [17]. A pool of the 4 libraries was selected in 2 steps repeated 3 times. In the first step, the libraries (adjusted to an OD 600 nm of 0.1) were treated with 25 µg/ml of histones during 3 h at 37°C with agitation in HAH medium (HBSS-, casamino acid 0.9% and HEPES 10 mM) containing Km. In the second, the recovery, step, we added one volume of LB 2X containing Km 100 µg/ml, CaCl_2_ 10 mM and MNase 40 U/ml (Fermentas) and grew the bacteria for 2 h at 37°C with agitation. Then, the bacteria were washed once in HAH medium prior the next selection with histones. We quantified living bacteria by plating an aliquot of a serial dilution series and determining the number of colony forming unit (CFU)/ml.

To obtain the plasmid population of the libraries, an aliquot of the bacterial suspension at the end of the selection was plated on LB Km agar plates. After an overnight incubation, the bacterial lawns were collected, pooled and frozen until the plasmids were isolated. Plasmid isolations were done with the HiSpeed Plasmid midi kit (Qiagen).

### DNA Microarrays

For each array, 3 µg of sample plasmid DNA was mixed with the following control plasmid DNA: 1000 ng pGIBS-DAP (ATCC#87486), 100 ng pGIBS-THR (ATCC# 87484), 10 ng pGIBS-TRP (ATCC# 87485) and 1 ng pGIBS-PHE (ATCC# 87483). The plasmid mixture was digested at 37°C overnight with 1 Unit each of *Alu*I and *Rsa*I (Invitrogen) in a reaction containing 50 mM Tris-HCl (pH 8.0), and 10 mM MgCl_2_. Reactions were heat inactivated at 70°C for 15 min. 10X One-Phor-All Buffer (Amersham Pharmacia Biotech, Piscataway, NJ) was added to the digestions to a final 1X concentration. 1 µL RQDNAse I (Fisher) was added to the reactions and incubated at 37°C for 2 min followed by heat-inactivation at 98°C for 20 min.

One µl of Exonuclease III (Fisher) was added to the reactions and incubated at 37°C for 15 min followed by heat inactivation at 98°C for 20 min. The resulting fragmented single stranded DNA was then labeled with biotinylated ddUTP using the Enzo BioArray™ Terminal Labeling Kit (ENZO Life Sciences, Farmingdale, NY) following the manufacturers’ protocol. Affymetrix *E. Coli* Antisense GeneChip® arrays (Affymetrix, Santa Clara, CA) were handled at the University of Colorado DNA Microarray Facility according to manufacturer’s specifications using a GeneChip® Hybridization oven,GeneChip® Fluidics Station, GeneArray® scanner and GeneChip® Operating Software 1.1 (Affymetrix). Raw microarray data can be found at ArrayExpress, accession number E-MEXP-3919.

### Microarray Data Analysis

Data analysis was completed by utilizing SCALEs software developed by Lynch *et al.* according to author’s instructions [17]. Signal values corresponding to individual probe sets were extracted from the Affymetrix data file and partitioned into probe sets based on similar affinity values. Background signal for each probe was subtracted according to conventional Affymetrix algorithms (MAS 5.0). Non-specific noise was determined as the intercept of the robust regression of the difference of the perfect match and mismatch signal against the perfect match signal. Probe signals were then mapped to genomic position as the Tukey’s bi-weight of the nearest 25 probe signals and noise was removed by applying a medium filter with a 1000 bp window length. Gaps between probes were filled in by linear interpolation. This continuous signal was decomposed using an N-sieve based analysis and reconstructed on a minimum scale of 500 bp as described in further detail by Lynch *et al*. [17]. Signals were further normalized by the total repressor of primer (ROP) signal, which is on the library vector backbone and represents the signal corresponding to the total plasmid concentration added to the chip.

### Cloning

All reagents, enzymes and kits were used according to manufacturer’s recommendations. The genomic DNA of *E. coli* K12 strain was used as template for high fidelity PCR (Phusion from Finnzymes) with the primers mention in Table 1. The PCR products were digested with restriction enzymes (from Fermentas) according to the restriction site present on the primers. The digested PCR product was inserted by ligation (T4 DNA Ligase from Fermentas) in pBAD18 (Km^R^) and pBAD33 (Cm^R^) vectors digested with the same restriction enzymes. The ligation products were introduced into *E. coli* TOP10 by heat shock transformation (Invitrogen) and selection was carried on LB agar with the appropriate antibiotic. After checking the inserts by sequencing, the plasmids were introduced into MACH1 for further analysis. The clones obtained are summarized in Table 2.

**Table 1:** Primers designed for the different clonings of the *wcaI/J* and *wzc/wcaA* fragments in pBAD33 and pBAD18 (see Fig. 2).

Name	Sequence	Restriction
wcaI_F02	GGGGTACCCCggagtgAAACG**ATG** AAAATACTGGTCTACGGC	*Kpn*I
wcaI_R01	AACTGCAGT**TTA**TCCCCGAATATCATTTATAAATTG	*Pst*I
cpsB_F02	GGGGTACCCCggagtgAAACG**ATG** GCGCAGTCGAAACTCTATCC	*Kpn*I
cpsB_R01	AACTGCAGT**TTA**CACCCGTCCGTAGCGATCC	*Pst*I
cpsG_F02	GGGGTACCCCggagtgAAACG**ATG** AAAAAATTAACCTGCTTTAAAGC	*Kpn*I
cpsG_R01	AACTGCAGT**TTA** CTCGTTCAGCAACGTCAGCAG	*Pst*I
wcaJ_F02	GGGGTACCCCggagtgAAACG**ATG** ACAAATCTAAAAAAGCGCGAGC	*Kpn*I
wcaJ_R01	AACTGCAGT**TCA** ATATGCCGCTTTGTTAACGAAACC	*Pst*I
wzc_F01	GGGGTACCCCggagtgAAACG**ATG**ACAGAAAAAGTAAAACAACATGCC	*Kpn*I
wzc_R01	GCTCTAGA **TTA**TTTCGCATCCGACTTATATTCG	*Xba*I
wcaA_F01	GGGGTACCCCggagtgAAACG**ATG**AAAAACAATCCGCTGATCTCAATC	*Kpn*I
wcaA_R01	GCTCTAGA **TTA**GCGCCCCCGAATACCATCAG	*Xba*I

On the sequence of the primers is represented: the restriction site (underlined); the Shine Dalgarno (lower case letter); start or stop codon (bold) for the forward (F symbol in the primer name) or reverse (R symbol) primers, respectively.

**Table 2:** bacterial strains and plasmids.

Strain	Plasmid	Reference
*Escherichia coli*		
MACH1-T1	pSMART-LCKm	
–	pBAD33 pBAD18	This study
–	pBAD33*cpsG* pBAD18*wcaI/cpsB*	–
–	pBAD33*wcaI* pBAD18*cpsB/cpsG*	–
–	pBAD18	–
–	pBAD18*wcaI/cpsB*	–
–	pBAD18*cpsB/cpsG*	–
–	pBAD18*wcaI*	–
–	pBAD18*cpsB*	–
–	pBAD18*cpsG*	–
–	pBAD18*wzc/wcaA*	–
–	pBAD18*wzc*	–
–	pBAD18*wcaA*	–
TOP10	pBAD33 pBAD18	–
–	pBAD33*wcaI/cpsB* pBAD18*cpsG/wcaJ*	–
–	pBAD33*cpsG/wcaJ* pBAD18*wcaI/cpsB*	–
–	pBAD33*cpsBcpsG* pBAD18*wcaI*	–
–	pBAD33*cpsB* pBAD18*wcaI*	–
–	pBAD33 pBAD18*wcaIcpsB*	–
–	pBAD33 pBAD18*cpsBcpsG*	–
–	pBAD33*cpsG* pBAD18*cpsB*	–
–	pBAD33*cpsB* pBAD18*cpsG*	–
–	pBAD33*wcaI* pBAD18	–
–	pBAD33*cpsB* pBAD18	–
–	pBAD33*cpsG* pBAD18	–
–	pBAD33*wcaJ* pBAD18	–
–	pBAD33*wcaJ* pBAD18*cpsB/G*	–
–	pBAD33*cpsG/wcaJ* pBAD18	–
–	pBAD33*cpsG* pBAD18*wcaJ*	–
*Klebsiella pneumoniae*		
Kp52 (52145)		[36]
52K10		[16]
52O21		[16]
*Shigella flexneri*		
M90T		[37]
M90T *rfbA-*		[24]
M90T *waaL-*		[24]

### Histone killing Assay

For MACH1 carrying pBAD33 and/or pBAD18 constructs were grown overnight in LB with antibiotics and L-arabinose 0.4%. *S. flexneri* and *K. pneumoniae* were grown in TSB and LB respectively for 3 h prior to the assay. Bacteria were harvested and resuspended in HAH medium. The bacterial suspensions were adjusted to a final concentration of 10^6^ bacteria/ml and co-incubated with different histone concentrations. The histone concentrations tested started from 100 µg/ml, or 250 µg/ml for *K. pneumoniae*, down to 0.625 µg/ml by serial dilution of ½. The surviving bacteria were enumerated by serial dilution in PBS MgCl_2_ 1 mM and plating. The bacterial enumeration determined the minimal bactericidal concentration of histones to kill 90% (MBC90) and 99% (MBC99) of the inoculum after 1 h of incubation.

### LPS Crude Extract Analysis

LPS was extracted from plate cultures by the proteinase K method [18]. LPS samples were separated by Tris-Tricine-sodium dodecyl sulfate (SDS)-polyacrylamide gel electrophoresis (gradient gel 10–20%, Criterion Biorad) as described by Lesse and colleagues [19]. The LPS was visualized by silver staining after periodate treatment [20].

### Exopolysaccharide Quantification

The exopolysaccharides of *E. coli* over-expressing the selected genes were extracted and purified from overnight liquid culture containing arabinose as previously described [21]. The culture was first boiled and after spinning down the bacterial remnants, the supernatant was precipitated with ethanol and then dialyzed. To quantify the concentration of exopolysaccharides, we determined the carbohydrate concentration (mg/ml) with the glycoprotein carbohydrate estimation kit (Thermoscientific).

## Results

### Positive Selection of Libraries of Histone Resistant *E. coli*

**Figure 1:**
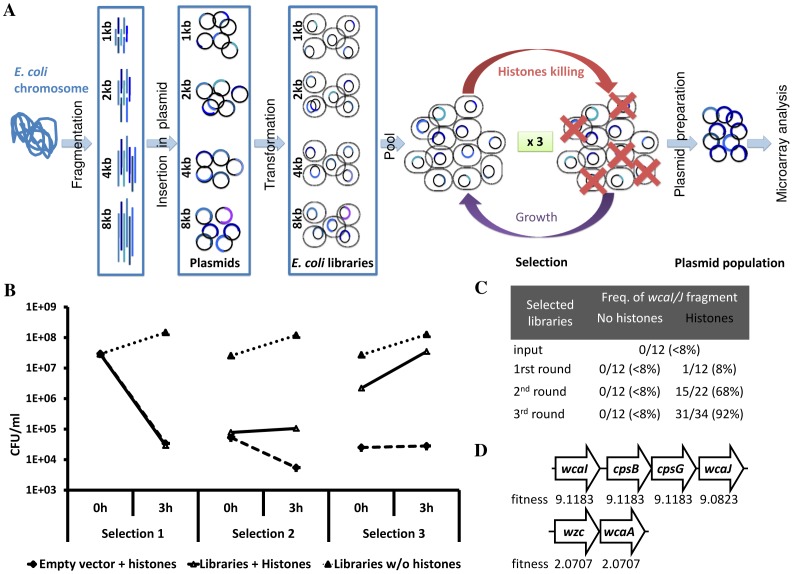
Screening of histone resistant *E. coli* from pooled over-expression libraries. [17]. The libraries consisted of a pooled of *E. coli* harboring plasmids carrying random fragments of *E. coli* DNA of 1, 2, 4 and 8 kb. The selection of the libraries consisted of elimination of histone susceptible clones (3 h), followed by a recovery step (2 h), and this repeated 3 times. After the selection, the plasmid population was extracted and analyzed by microarray. (B) During the selection process, the bacterial population was quantified by serial dilution and plating. Two controls were running in parallel, one consisting of the libraries without histone selection, and another of *E. coli* carrying empty vector submitted to histone killing. (C) Several clones from the different steps of the selection were isolated to identify the plasmid insert by sequencing. An insert corresponding to the genomic region from *wcaI* to *wcaJ* was enriched during the histone selection. The *wcaI/J* region is part of the colanic acid cluster. (D) After microarray analysis of the histone selected population, two plasmid inserts showed up belonging to the colanic acid cluster: *wcaI/cpsB/cpsG/wcaJ* and *wzc/wcaA*. The fitness for each gene was calculated by the logarithmic value of the intensity of the DNA fragment divided by the logarithmic value of the vector’s backbone and compared to the normalized intensity before selection.

To determine mechanisms responsible for histone resistance, we set up a screen based on positive selection of *E. coli* libraries, which over-express random genomic fragments [17]. We used a pool of four libraries consisting of *E. coli* carrying plasmid harboring random fragments of the *E. coli* K12 genome (Fig. 1A). Each library covers the entire *E. coli* genome in 1, 2, 4 and 8 kb fragments allowing us to screen the genome several times and, depending on the size of the fragment, refine the region conferring the expected phenotype [17]. Thus, the selection was designed to enrich histone resistant clones. Because *in vivo* bacteria encounter more than one kind of histone, we used a mixture of histones isolated from calf thymus. Prior to the selection, we tested the antimicrobial activity of the whole histones as provided by the manufacturer or after re-purification by chromatography. The re-purification step did not change the dose or kinetics of *E. coli* killing (data not shown). Because of the depletion of histones H1 by re-purification, we used the whole histones from the same batch for further experiments. We incubated the libraries with 25 µg/ml of histones for 3 h interrupted by a recovery of the culture by incubation in Luria Broth (LB) in presence of Micrococcal nuclease (MNase) for 2 h (Fig. 1A) and repeated the procedure 3 times. The MNase treatment was necessary to disperse bacteria that aggregated due to the released of DNA. We monitored the selection process by quantifying the number of colony forming units (CFU) and by isolating clones to identify their plasmid inserts before and after each histone selection. In parallel to the selection we ran two controls. The first one was to confirm that *E. coli* is susceptible to histones under the experimental conditions. To achieve this we incubated *E. coli* carrying the empty vector in the same conditions as the selected libraries. In the second control we measured the growth of the *E. coli* libraries incubated in the same experimental conditions but in absence of histones, to determine the growth in the absence of selection. The bacterial suspensions incubated with histones showed fewer CFU/ml than the control cultures after the first recovery step although we washed and adjusted the suspensions to the same OD at each step. This difference confirms that histones were bactericidal (Fig. 1B). Notably, at the beginning of the third histone selection, the CFU/ml of the selected libraries was higher than before the second selection, indicating that fewer bacteria were dead at the beginning of the third selection. There were significantly more survivors in the pooled libraries than in *E. coli* carrying the empty vector when incubated with histones (Fig. 1B), confirming a positive selection of histone resistant bacteria.

After each selection we isolated clones from the pooled libraries incubated in the presence and in the absence of histones. The 10 clones isolated from the second selection were more resistant to histones than the 10 clones isolated from the libraries that were not under selection pressure (See Fig. S1). We sequenced 500–1000 bp of the 5′ and 3′ of their plasmid inserts, and, interestingly, a fragment including the *wcaJ/I* region (from genomic position 2 118 398 to 2 123 285 in the *E. coli* K12 chromosome, positions given by the Colibri genolist website) was enriched after each histone selection. About 90% (31/34) of the clones isolated after the last round of selection contained this fragment (Fig. 1C), indicating a high selectivity.

Thus, the positive selection of the pooled libraries was confirmed by: (i) the increasing bacterial concentration of the libraries after incubating them with histones, (ii) the increase in histone resistance of clones isolated from the libraries, and (iii) the enrichment of one particular plasmid insert in clones isolated from the selected libraries.

We were interested both in the most abundant as well as in rarer fragments that contribute to histone resistance. Hence, we compared the plasmid population from the initial and selected pooled libraries by microarray analysis. As a control, we confirmed that there was no selection in the sample obtained from control libraries that were not exposed to histones through the experiment. In contrast, 8 kb fragments carrying the *wcaJ, cpsB, cpsG and wcaI* genes were strongly enriched after histone selection. This data is in agreement with the sequencing data presented in Fig. 1C.

The genes present in the *wcaJ/I* fragment belong to the colanic acid cluster. Interestingly, in the microarray analysis we also found the *wzc/wcaA* fragment, which also belong to the colonic acid cluster, albeit at lower intensity (Fig. 1D). The intensity of a spot in microarray was normalized by the logarithmic value of the intensity of the spot corresponding to the DNA fragment divided by the logarithmic value of the intensity for the vector’s backbone (see Materials and Methods). Then, the fitness attributed to a spot or gene was calculated by comparing the intensities before and after selection. Overall data of the fitness are in Table S1. On average, the *wcaJ/I* fragment had fitness of 9.1 in comparison with 2.1 for the *wzc/wcaA* fragment (Fig. 1D).

### Cloning Under Arabinose Promoter and Toxicity of Over-expressed Genes

**Figure 2:**
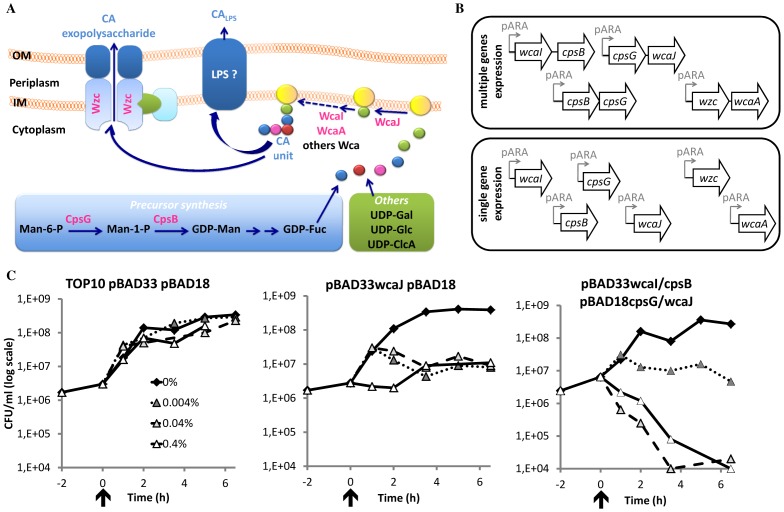
Involvement of the selected genes in the colanic acid pathway and cloning under arabinose promoter. (A) The genes screened for histone resistance (bold pink) are represented in the scheme of the colanic acid pathway. The first steps of the colanic acid pathway consist on the production of activated sugar in the cytoplasm of the bacterium. Mannose-6-phosphate (Man-6-P) leads to the production to GDP-fucose by CpsG and CpsB. Others activated sugars, UDP-galactose (UDP-Gal), -glucosamine (-Glc) and –glucuronic acid (-GlcA), are produced also for other cellular components. The assembly of the first sugar unit, colanic acid (CA) unit, is assembled on the lipid carrier, called undecaprenyl pyrophosphate, by Wca proteins, such as WcaJ, WcaI and WcaA. Then the CA unit is exported and polymerized by the Wzc machinery, for the production of the exopolysaccharide. Another possibility is the addition of CA unit on the short form of the lipopolysaccharide (LPS) by an unknown mechanism [21], [22]. (B) The selected genes belonging to the colanic acid cluster were cloned under arabinose promoter on pBAD33 and pBAD18 vectors. Different constructs were obtained to address different gene combinations in the histone resistance and to refine the essential genes for the resistance. (C) The toxicity of the arabinose-induced genes was tested by following the bacterial population (CFU/ml). After 2 h of culture (indicated by an arrow and considered as time 0), L-arabinose was added at 0.4 (open triangle, full line), 0.04 (light gray triangle, dash line) and 0.004% (dark gray triangle, dot line) final concentration or not (black diamond, full line), and the culture was prolonged during 6 h.

The colanic acid cluster encodes genes required for the production of exopolysaccharide and for semi-smooth LPS form [21], [22]. Intriguingly, we did not select all the genes of the cluster (Fig. 2A). Instead, we selected for enzymes involved in, (1) the synthesis of sugar precursor in the cytoplasm, CpsB and -G, (2) the assembly of the sugar unit at the inner membrane by the glycosyltransferases (cytoplasmic side), WcaJ, -I and –A, and (3) the export of the polysaccharide, Wzc.

To determine which genes in this cluster were required to confer histone resistance, we cloned all the genes in vectors either alone or in groups as illustrated in Fig. 2B. We cloned the genes under the arabinose promoter, which allows the tight regulation of their expression. We also designed and generated different combination of genes on two different vectors, pBAD33 (Cm^R^) and pBAD18 (Km^R^), to test different genes combination by introducing two plasmids carrying different constructs in one *E. coli* strain (Fig. 2B, Table 2).

We first tested whether over-expressions of the different genes were toxic. After inducing gene the expression with arabinose we observed three different phenotypes. First, over-expression did not influence bacterial growth (Fig. 2C panel 1). This was observed on the different induced constructs in TOP10 carrying empty vectors, pBAD18 pBAD33, or plasmids carrying inserts: pBAD18*wcaI* pBAD33*cpsBcpsG;* pBAD18*wcaI* pBAD33*cpsB;* pBAD18*wcaIcpsB* pBAD33*;* pBAD18*cpsBcpsG* pBAD33*;* pBAD18*cpsB* pBAD33*cpsG;* pBAD18*cpsG* pBAD33*cpsB*; pBAD18 pBAD33*wcaI*; pBAD18 pBAD33*cpsB*; pBAD18 pBAD33*cpsG*. Second, we observed an inverse correlation between the arabinose concentration and bacterial growth (Fig. 2C panel 2). Four clones fall in this category: pBAD33*wcaJ* pBAD18; pBAD33*wcaJ* pBAD18*cpsB/G*; pBAD33*cpsG/wcaJ* pBAD18; or pBAD33*cpsG* pBAD18*wcaJ*. Third, over-expression was toxic (Fig. 2C panel 3). This was observed for clones TOP10 pBAD33*wcaI/cpsB* pBAD18*cpsG/wcaJ* and TOP10 pBAD33 *cpsG/wcaJ* pBAD18 *wcaI/cpsB*. Expression of *wcaJ* was the only one that was bacteriostatic and, its expression in combination with other selected genes, became bactericidal. Furthermore, both the microarray and sequencing analysis of the selected clones showed a truncation at the 3′ end of the *wcaJ*. Because of the selection of the truncated *wcaJ* and the toxicity of the full length gene when overexpressed, we excluded *wcaJ* for further analysis.

### Contribution of the Colanic Acid Cluster Genes to Histone Resistance

To determine the contribution of the different genes to histone resistance, we determined the minimal bactericidal histone concentration to kill 90 (MBC90) or 99% (MBC99) of the bacterial inoculum in 1h after inducing over-expression with arabinose.

**Figure 3:**
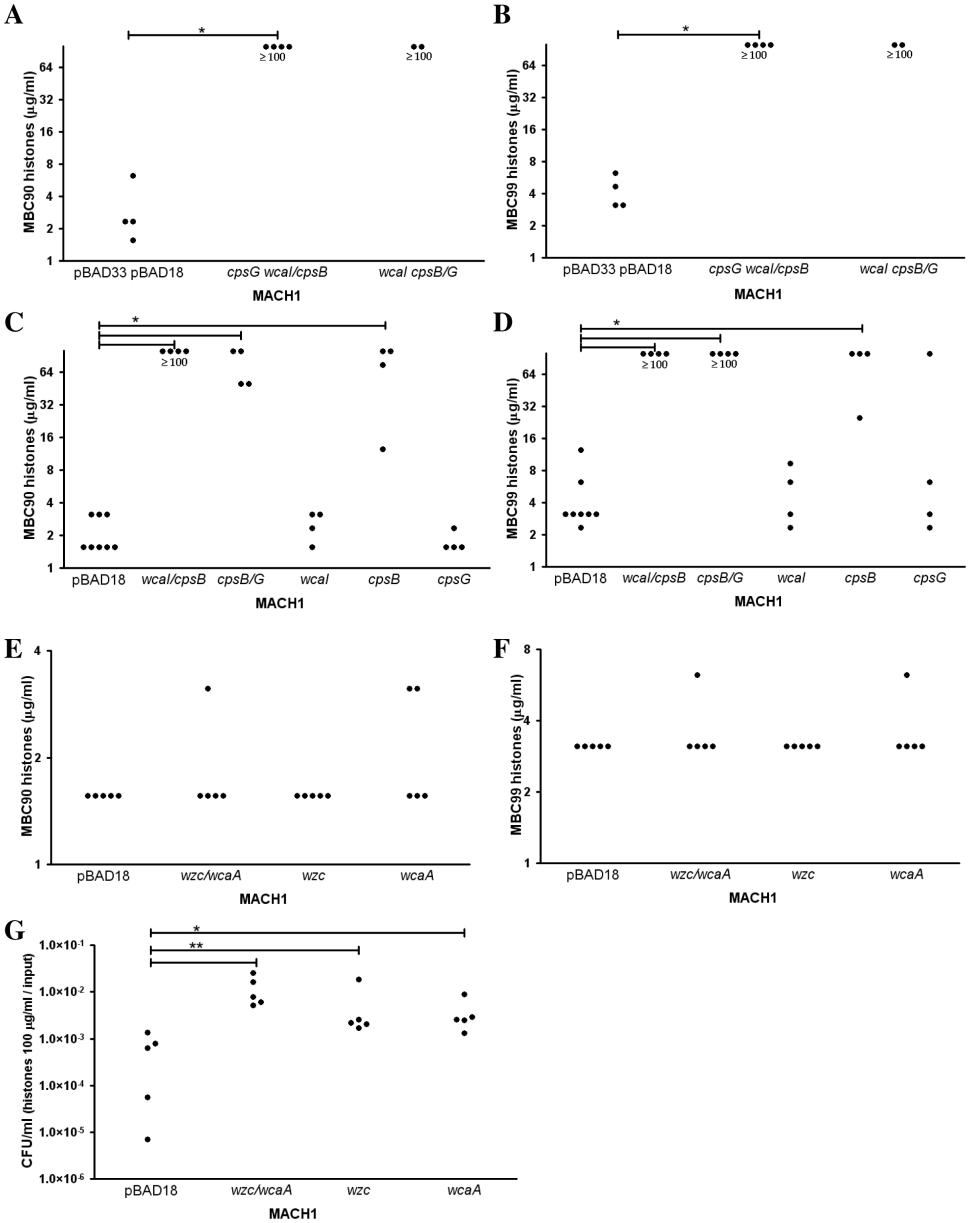
Impact of over-expression of colanic acid related genes identified in the screen on the histone resistance. We determined the minimal bactericidal concentration of histones to kill 90% (MBC90, left panels) or 99% (MBC99, right panels) of the initial bacterial inoculum after 1 h of incubation. (A–B) The simultaneous over-expression of *wcaI*, *cpsB*, *cpsG* genes in MACH1 carrying the plasmids pBAD33*cpsG* pBAD18*wcaI/cpsB* or pBAD33*wcaI* pBAD18*cpsB/cpsG* led to a high histone resistance in comparison with the strain carrying the empty vectors. (C–D) To refine the genes contributing the resistance, we tested the over-expression of *wcaI/cpsB*, *cpsB/cpsG,* or single genes, *wcaI*, *cpsB*, *and cpsG,* carried by pBAD18. (E–F) The other set of genes related to the colanic acid, *wzc/wcaA*, did not show any significant histone resistance in term of MBC90 and MBC99 in comparison with the bacteria carrying the empty vector, while the genes were over-expressed together (MACH1 pBAD18*wzc/wcaA*) or individually (MACH1 pBAD18*wzc* and MACH1 pBAD18*wcaA*). (G) The *E. coli* over-expressing this different combination of *wzc* and *wcaA* genes showed a higher number of survivors after 1 h treatment with histones at 100 µg/ml. To be able to compare the different clones, we standardized the values by dividing the CFU/ml after 1 h treatment by the CFU/ml of the inoculum. Each dot represent the result of one experiment and at least three independent experiments have been done. The differences were considered significant by the Mann-Whitney test with p≤0.05 (*) and p≤0.01 (**).

Over-expressing *wcaI*, *cpsB* and *cpsG* genes, in *E. coli* MACH1 resulted in high resistance to histones (MBC99 and 90≥100 µg/ml) (Fig. 3A–B). These results validated our screening. Over-expression of two genes *wcaI/cpsB* or *cpsB/cpsG,* or *cpsB* alone also led to a high histone resistance (Fig. 3C–D) showing that *cpsB* is mainly responsible this phenotype. Interestingly, *cpsB* in combination with *wcaI* or *cpsG* conferred even a higher resistance as showed by the MBC90 (Fig. 3C) indicating synergy between these genes.

*wzc/wcaA* was the second fragment related to colanic acid identified in the screen. The MBC90 and 99 of the over-expression of *wzc* and *wcaA* or either one of those genes alone did not differ from the empty vector control (Fig. 3E–F). Intriguingly, the survival to a 1 h exposure to 100 µg/ml of histone was better in bacteria over-expressing *wzc* and *wcaA* or either gene alone than in cells carrying an empty vector (Fig. 3G). This low resistance to histone correlated with the low frequency of selection of these genes in our screen (Fig. 1D).

### Clones Over-expressing Genes in the Colonic Acid Pathway have a Normal Outer Membrane Barrier Function and Produce O-antigen as well as Exopolysaccharide

The resistance to histone of clones overexpressing genes involved in colonic acid synthesis could be explained by alteration in the barrier function of the outer membrane. With this in mind, we tested the susceptibility of different clones to detergents (sodium deoxycholate and SDS) and antibiotics (vancomycin, bacitracin and novobiocin). None of the clones tested showed any significant differences in comparison with the clone carrying the empty vectors (data not shown).

**Figure 4:**
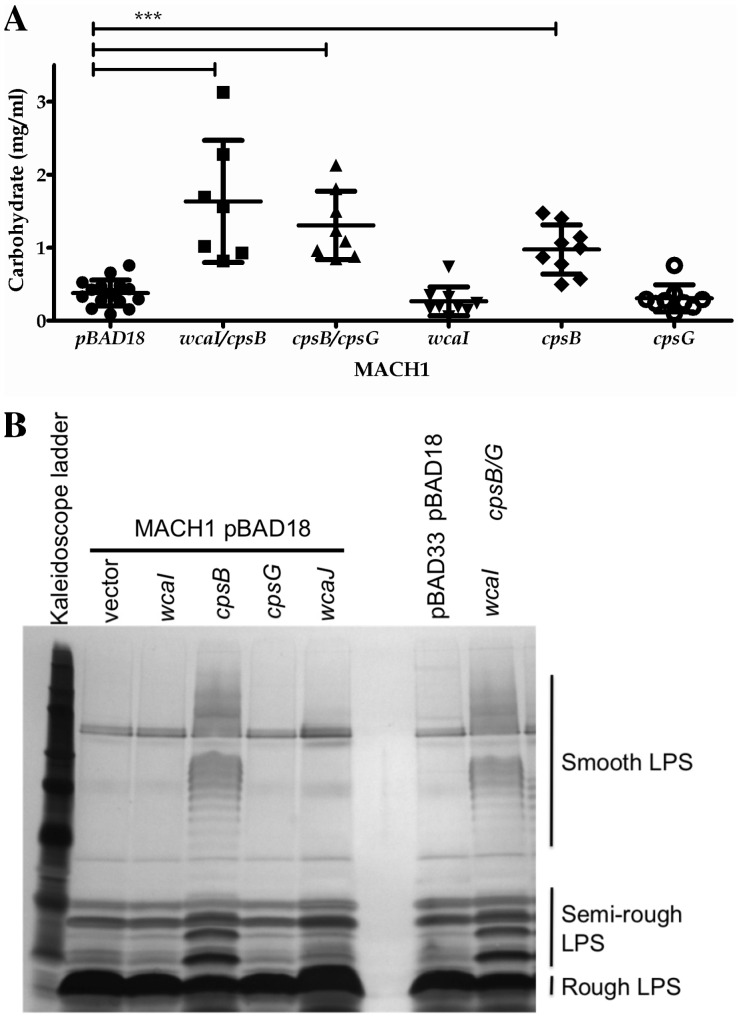
Characterization of exopolysaccharide and lipopolysaccharide production in *E. coli* MACH1 expressing *wcaI, cpsB* and *cpsG* genes. (A) We purified the exopolysaccharide from supernatants of overnight liquid culture *E. coli* MACH1 pBAD18 empty vector or carrying *wcaI/cpsB*, *cpsB/cpsG*, *wcaI*, *cpsB* or *cpsG*, growing in presence of arabinose. The isolated exopolysaccharide was quantified by determining the carbohydrate content. Each dot represents the result of one measurement. The differences were considered significant by the Mann-Whitney test with p≤0.001 (***). (B) From the same liquid cultures, an aliquot was taken to analyze the LPS. The preparations of proteinase K-treated whole cell lysates were run on a Tris-tricine polyacrylamide gel and silver stained after periodate treatment. The gel represents an example of 3 independent experiments.

The proteins encoded by the colanic acid cluster are responsible for the production of the exopolysaccharide colanic acid ([22], Fig. 2A). In *E. coli* K-12 strain MG1655, this cluster can also contribute to the production of a short form of an O-antigen [21]. We studied the production of exopolysaccharide and LPS in clones over-expressing the selected genes. The exopolysaccharide was extracted and isolated by differential precipitation steps. We failed to detect fucose colorimetrically in the extracts, as a measure of colanic acid [23]. Instead, we quantified the exopolysaccharide through its carbohydrate content. MACH1 strains over-expressing *wcaI/cpsB*, *cpsB/cpsG,* or *cpsB* had more exopolysaccharide than strain carrying the empty vector (Fig. 4A).

We also analyzed the LPS of proteinase K-treated whole cell lysates by separation on polyacrylamide gels. *E. coli* MACH1 carrying empty vector(s) showed mainly a rough LPS, consisting on lipid A and the core. The over-expression of *wcaI/cpsB/cpsG* or *cpsB* led to the production of a LPS of higher molecular weight (visible by the typical ladder pattern in the higher part of the gel), called semi- and smooth LPS and corresponding to the presence of different length of the O-antigen attach to the lipid A – core domains (Fig. 4B). Thus, the over-expression of *cpsB* is the key factor for the production of an exopolysaccharide and an O-antigen+ LPS, which could explain the histone resistance.

### The O-antigen of *K. pneumoniae* and *S. flexneri* Protects Against Histone Bactericidal Activity

**Figure 5:**
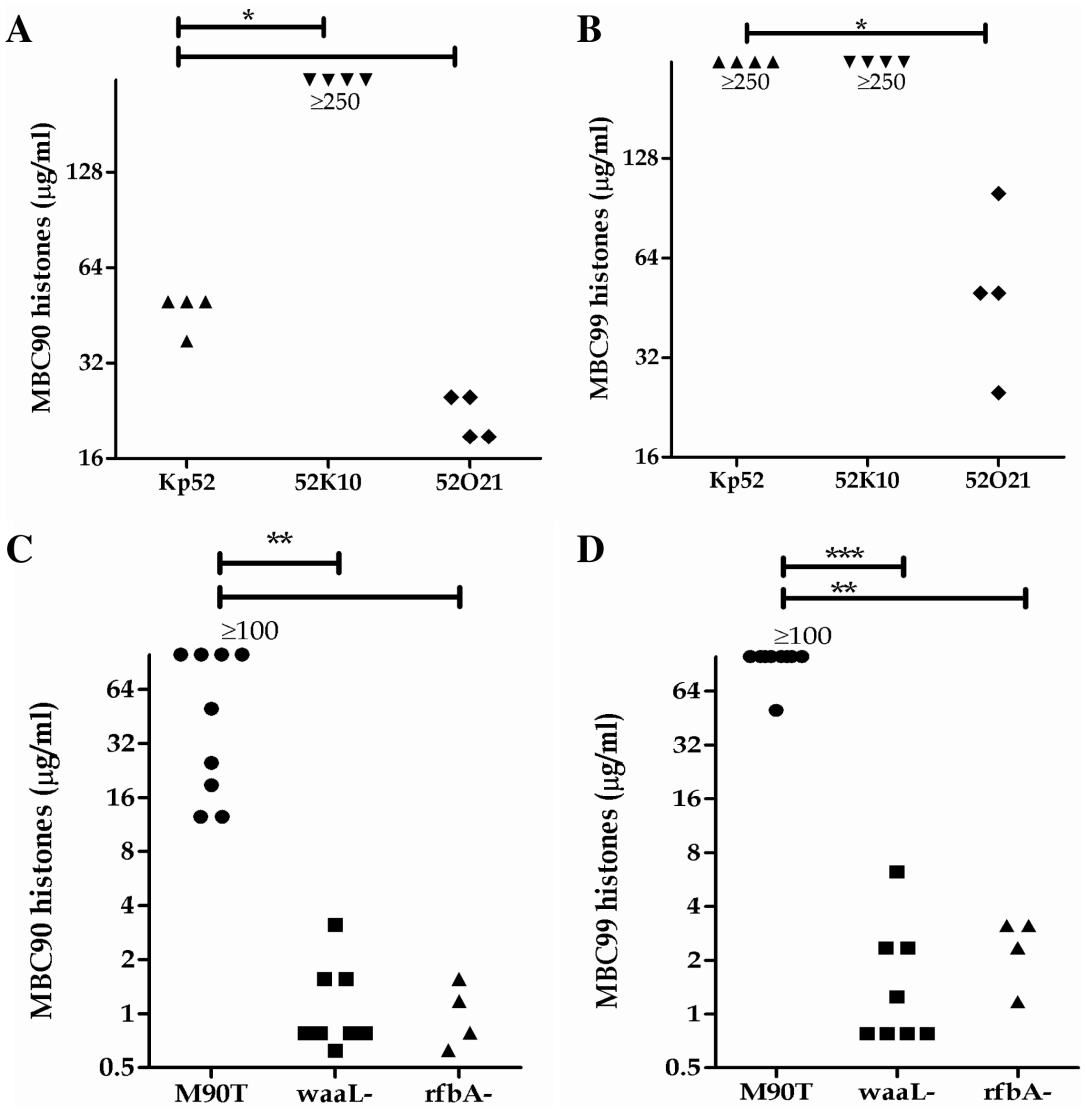
Contribution of capsule and LPS in histone resistance in *Klebsiella pneumoniae* and *Shigella flexneri*. MBC90 and MBC99 have been determined for (A–B) *K. pneumoniae* wild type (Kp52), acapsulated mutant (52K10) and O-antigen mutant (52O21), and (C–D) in *S. flexneri* background: wild type (M90T) and O-antigen mutant (*waaL-*). Each dot represents the result of one experiment and at least 4 independent experiments have been done. The differences were considered significant by the Mann-Whitney test with p≤0.05 (*).

We determined the contribution of exopolysaccharide and LPS to histone resistance and in the pathogenic Gram-negative bacteria, *S. flexneri* and *K. pneumoniae*. Both of these species produce O-antigen+ LPS but only *K. pneumoniae* is a capsulated. We compared the MBC90 and MBC99 of wild type *K. pneumoniae* Kp52145 strain with the acapsulate mutant 52K10 [16]. *K. pneumoniae* with or without capsule were resistant to histones (Fig. 5A), but the MBC90 of the acapsulate mutant is significantly higher than that of the wild type strain. The capsule seemed to have a limited impact on histone resistance. *S. flexneri* is naturally acapsulated but O-antigen+, showed a higher histone resistance than MACH1 *E. coli* carrying empty vectors (Fig. 5B, Fig. 3A–F). To address histone protection *via* the O-antigen, we compared the wild type *S. flexneri* strain M90T with a *waaL* and a *rfbA* isogenic mutants (O-antigen-, [24] and manuscript in preparation: Geldmacher *et al.*), as well as the wild type *K. pneumoniae* with the O-antigen mutant 52O21 [16]. The O-antigen- mutants in *S. flexneri* and *K. pneumoniae* backgrounds showed a significant higher sensitivity toward histones than the wild type strains (Fig. 5A–D). This last observation strongly suggests that the O-antigen is the main cell surface component to protect Gram-negative bacteria against histones.

## Discussion

### Rationale of Using Over-expression Libraries

Understanding how bacteria become more resistant or susceptible to AMPs can reveal their mode of action. One way to find out the mechanism of action of an AMP is to select susceptible clones to AMP(s) out of a mutant library. This approach has lead to a few interesting observations. For example, DltA, a protein that alanylates lipoteichoic acid in the cell wall of the opportunistic Gram-positive pathogen *Staphylococcus aureus*, confers resistance to cationic AMPs [25]. Another example suggested that the neutralization of phosphatidylglycerol by lysine, mediated by *mprF,* also leads to AMP resistance [26]. Using a different AMP, Li *et al.* proposed that lipid modifications, dependent on *dak2,* also change the bacterial susceptibility [27]. It is likely that AMP target essential bacterial components, negating the use of this type of selection. Furthermore, like in any screen, if several genes have the same function, deletion only one of them has little, if any, impact on the phenotype. Taking essential genes and gene redundancy into consideration, we chose a different approach. We selected clones from homologous libraries of *E. coli* harboring low copy plasmids carrying genomic DNA fragment, where the genes are expressed under their native promoters. By having the genes under their native promoter but in several copies, the gene(s) would be over-expressed only slightly because of the low copy number plasmid and, since they are under their original promoter, physiologically regulated.

### Advantages of Selecting Resistant Clones

The underlying hypothesis of a positive selection is that, in a susceptible bacterium, AMPs interact with their target(s) at a certain molar ratio. Over-expression of the target(s) makes the bacterium resistant to the AMP. A parameter to consider is the efficacy of the AMP action. Histones, at MBC90 and over, kill *E. coli* efficiently in less than 10 min. (Data not shown, [5]). Even though the bactericidal action is fast, it could induce bacterial response at a transcriptional level. Thus, over-expression libraries contain clones which should be already resistant even in the absence of the AMP, and some which would become resistant by regulation on the native promoter of the gene(s). Experimentally, a selection of resistant clones allows also a reliable and fast read out because the interesting clones will survive or even grow in the presence of the AMP, as we could observed while selecting with histones. Our selection procedure was validated by several controls. One set of controls was to follow the bacterial survival of the library in the presence of absence of the selection pressure, as well as the incubation of bacteria carrying the empty vector in presence with histones.

### Suitable Screening Approach for AMPs

The different histones could have different mode of actions. For example, histone H1 shows different biochemical properties than the core histones (pI, sequence, structure) suggesting a distinct bactericidal mechanism than the others histones. Nevertheless, we chose to perform our screen with mixture composed of 5 histones because *in vivo* bacteria are likely to encounter a complex variety of proteins. Furthermore, the histones we used are post-translational modified increasing the diversity of the proteins.

### Toxicity Due to the Over-expression of *wcaJ*

WcaJ is predicted to be the glycosyltransferase which loads the first sugar of what will become a polysaccharide on a lipid carrier, the undecaprenyl pyrophosphate ([22], Fig. 2A). This lipid carrier is a common transporter for precursors of essential bacterial cell wall components, such as LPS and peptidoglycan [28]. High amount of WcaJ in the bacterium might unbalance the use of the lipid carrier and the production of polysaccharide synthesis. The decrease of peptidoglycan synthesis could inhibit bacterial growth or be lethal to the bacterium, as we observed when over-expressing *wcaJ*. The co-expression of other downstream enzymes in the synthesis of the polysaccharide, such as WcaI, would increase the production of polysaccharide and induce bacterial lethality. This could explain our observation on the bactericidal effect of concomitant over-expression of the *wcaI/cpsB/cpsG/wcaJ* genes.

Moreover, it is known that growth rate, and in particular the presence of a strong proton motive force, can have an influence on the relative toxicity of different antibiotics, including AMPs [29]. In our studies, this may have played a role in the observed protection of slow growers, even though, the histone bactericidal activity is not affected by bacteriostatic conditions (unpublished data). However, in the case of over-expressing *cpsB* alone or in combination with *cpsG* or *wcaI*, we did not observe decreased in growth rate, and did confirm the production of an O-antigen thus suggesting that reduced growth rate did not play a major role in our observed protection.

### Capsule and Histone Killing

We selected genes involved in the production of the colanic acid in *E. coli* MG1655. In the over-expressing *E. coli* MACH1 clones, we quantified a significant production of an exopolysaccharide based on a carbohydrate determination. But in the different exopolysaccharide extracts we could not detect fucose using the sulfuric acid reaction, which is one of a typical sugar for the colanic acid exopolysaccharide. We can speculate that the over-expression of *cpsB* restored the LPS and exopolysaccharide pathways in *E. coli* MACH1.

Since we selected genes required for the production of an exopolysaccharide, we hypothesized that a bacterial capsule should also play a barrier role for histones. The capsule of *K. pneumoniae* prevents killing by human neutrophil alpha-defensin 1, polymyxin B or protamine, but not to complement activity [30]. Surprisingly, in our assays, the capsule of *K. pneumoniae* is not protective towards histones. The capsule may just delay the interaction with the bacterial cell wall but does not abrogate it. We could speculate that the absence of the capsule, which is not truly a barrier for histones, unmasked the O-antigen possessing better properties to interfere with the interaction of histones with the bacterial membrane.

### Interaction of Histones with Bacterial LPS

The first step of cationic AMPs, probably including histones, is to interact with the bacterial surface *via* electrostatic interaction [2], [31], [32]. Modifications of surface components for Gram-negative and -positive bacteria have been described to protect pathogens against a variety of AMPs by abrogating their interaction with the bacterial surface [2], [33]. One example of LPS modification is the addition of 4-amino-4-deoxy-l-arabinose on the lipid A, target of polymyxin [34]. This is consistent with our results, where we selected for genes related to O-antigen and exopolysaccharide synthesis. The O-antigen neutralized the negative charge of the bacterial surface decreasing the attractive properties toward cationic AMPs [2], [33]. In the case of *S. flexneri*, we showed that the presence of the O-antigen lead to the neutralization of the bacterial surface (manuscript in preparation: Geldmacher *et al.*). Moreover, it has been shown that histones interact with the lipid A *in vitro*, portion embedded in the outer membrane of Gram-negative bacteria [35]. Thus, we could speculate that the O-antigen could mask the lipid A and avoid the binding of histones on it.

To conclude, the electrostatic interactions of histones to the bacterial cell wall would be the first step of their mode of action, which will be common to Gram-negative and -positive bacteria. We could also speculate that the binding of histone to the lipid A of the LPS would lead to death of Gram-negative bacteria. It has been shown that LPS protect bacteria towards different AMPs [2], [33]. Moreover, these bacteria may have been confronted by a variety of histones present in intact or partial forms during their evolution or in a more recent history. Thus it is reasonable that histone protection may have played a role in the selection of current LPS properties.

## Supporting Information

Figure S1 **Growth curves of** ***E. coli*** **clones pre-incubated with 100, 25, 10 and 0 µg/ml final of calf thymus histones (annotated H100, H25, H10 and H0 and represented by a pink, red, orange and blue curves, respectively).** Single clones isolated during histone selection were tested for their histone resistance or susceptibility. As controls, we tested 10 *E. coli* clones isolated from the non-selected libraries and *E. coli* MACH1 pSMART-LCKm (empty vector). The bacterial suspensions were adjusted to final concentration of 10^6^ bacteria/ml in HAH medium (HBSS-, casa-amino acid 0.9% and HEPES 10 mM) supplemented with Km 50 µg/ml. The clones were incubated with 100, 25, 10 and 0 µg/ml final of calf thymus histones 1 h at 37°C with agitation. Then, the bacterial growth was allowed by adding 1 volume of 2X LB, CaCl_2_ 10 mM and Km 50 (37°C with agitation). The growth was followed in 96 well-plate by reading the absorbance at 600 nm during 10 h with a reading every 5 min. The growth curves would be dependent of the amount of living bacteria at the end of the incubation with histone, as shown by the profiles of the MACH1 pSMART-LCKm and the isolated clones from the non-selected libraries. The 10 clones isolated from the selected libraries showed higher resistance. SL2+ corresponds to the 1 to 10 clones isolated from the histone-selected libraries. SL2- corresponds to the 1 to 10 clones isolated from the non-selected libraries. MACH1 pSMART-LCKm is the control of *E. coli* harboring the empty vector.
(PDF)

Table S1 **Data from the microarray analyses.** The genes are ranked in function of their fitness as described in the manuscript.
(XLSX)
