# Supplementary figures and images for: O-Antigen Protects Gram-Negative Bacteria from Histone Killing
Source: PLoS One. 2013 Aug 8;8(8):e71097. doi: 10.1371/journal.pone.0071097 (PMC3738592; doi:10.1371/journal.pone.0071097)

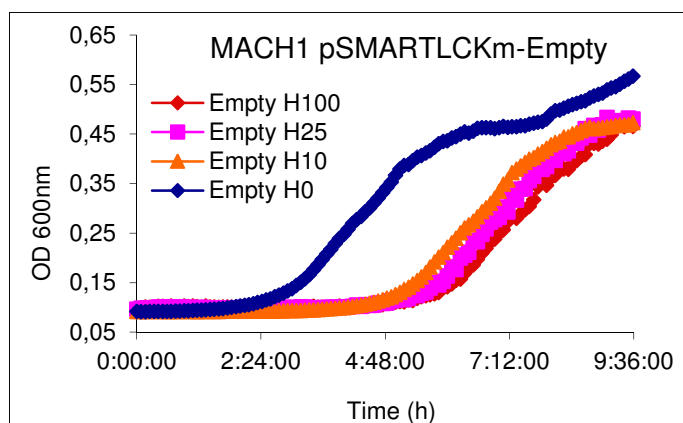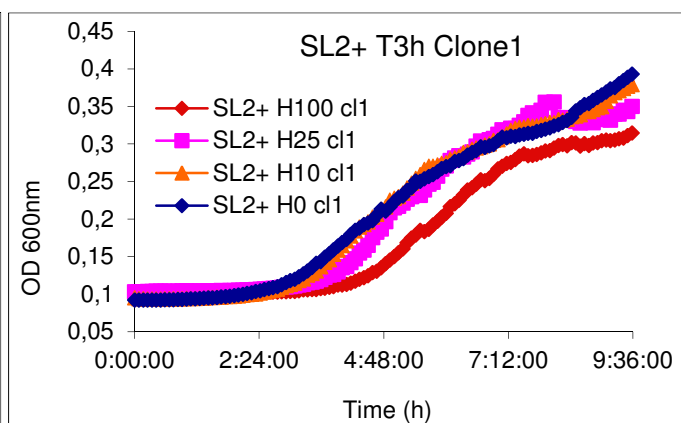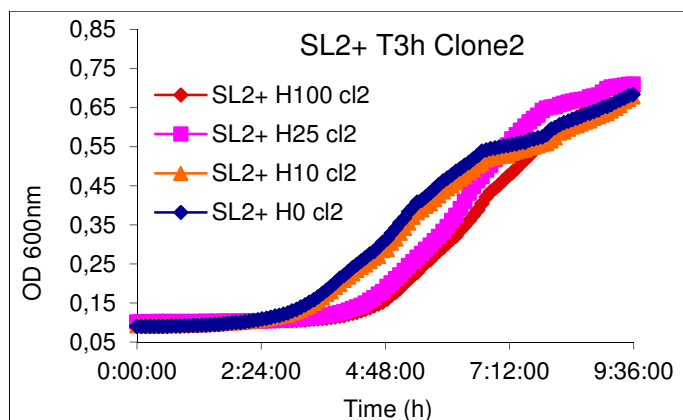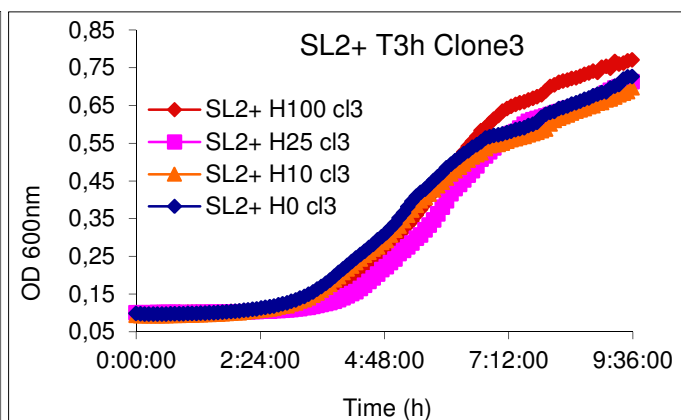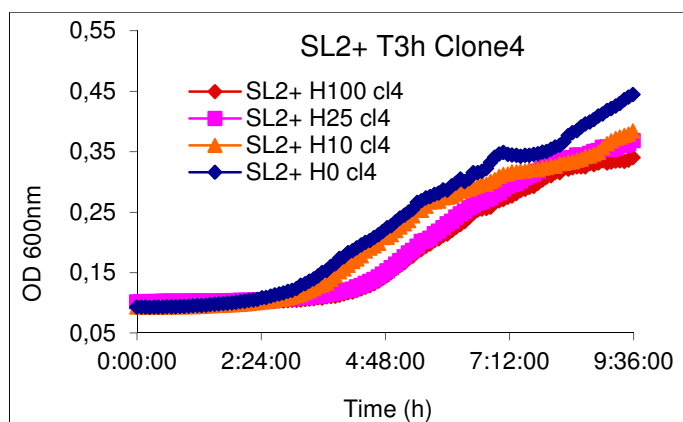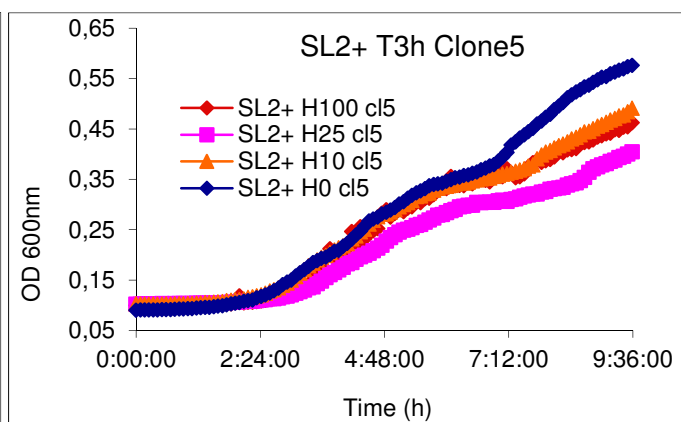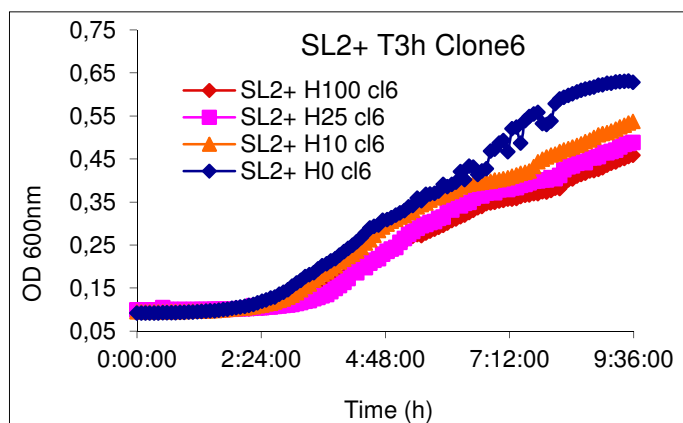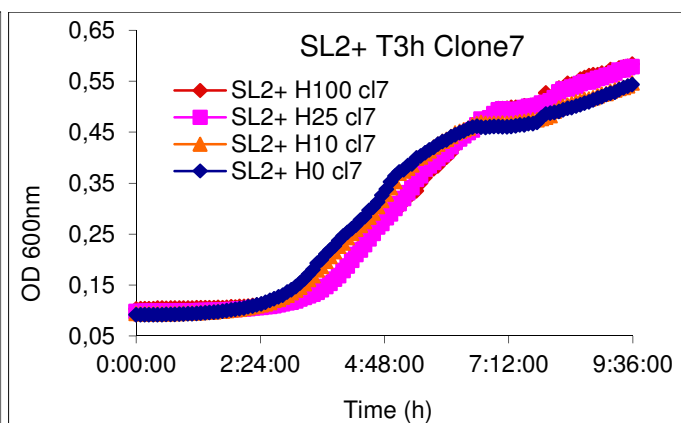

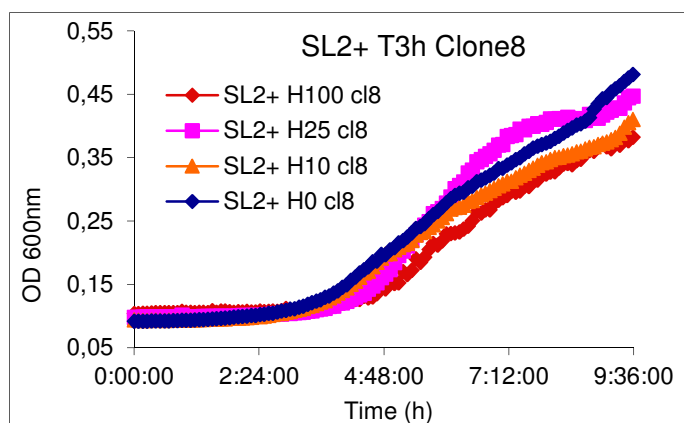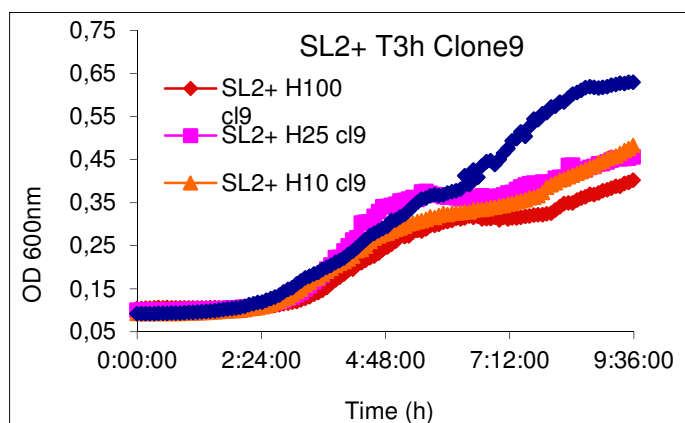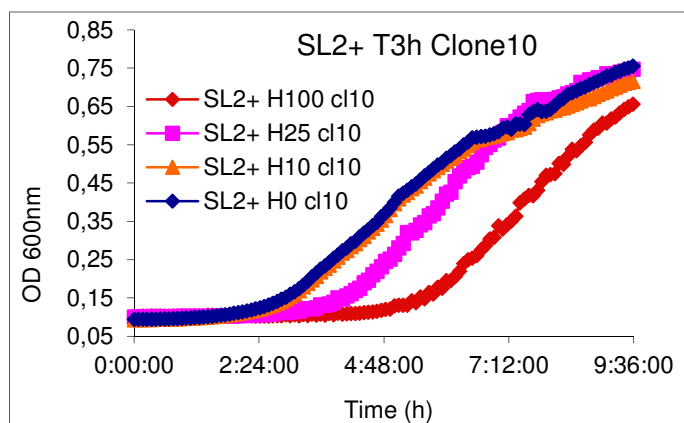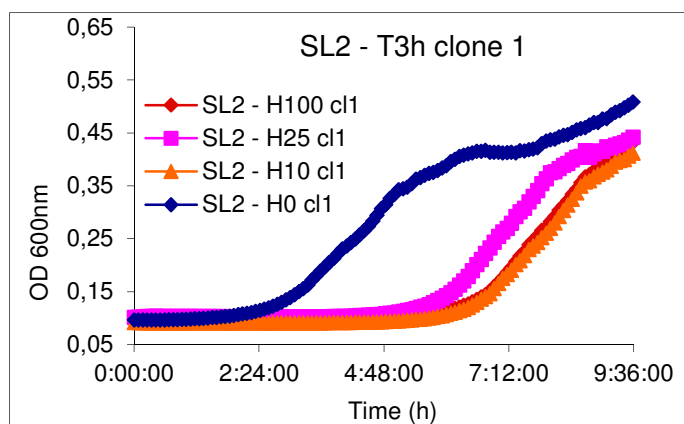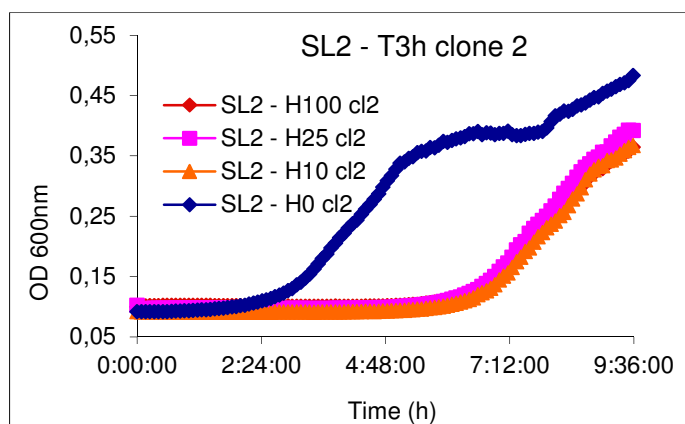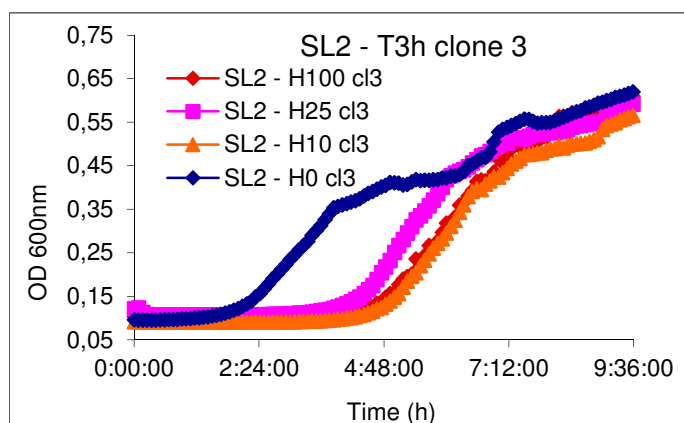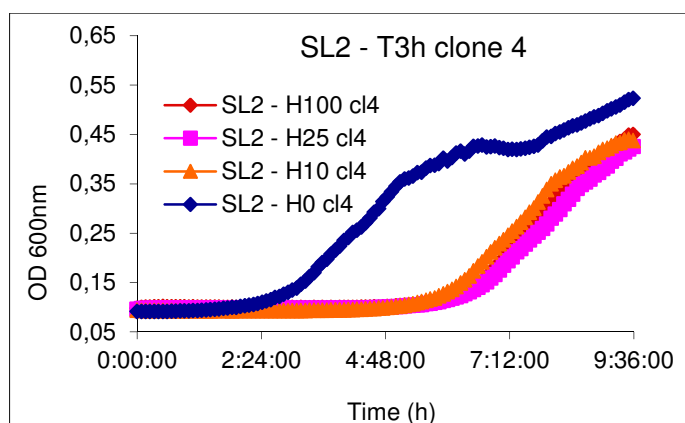

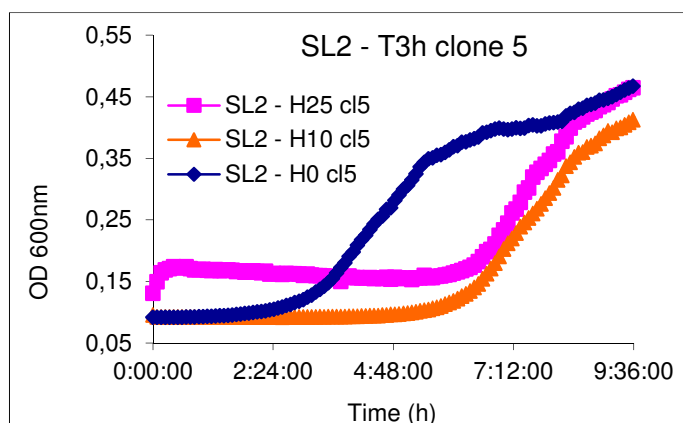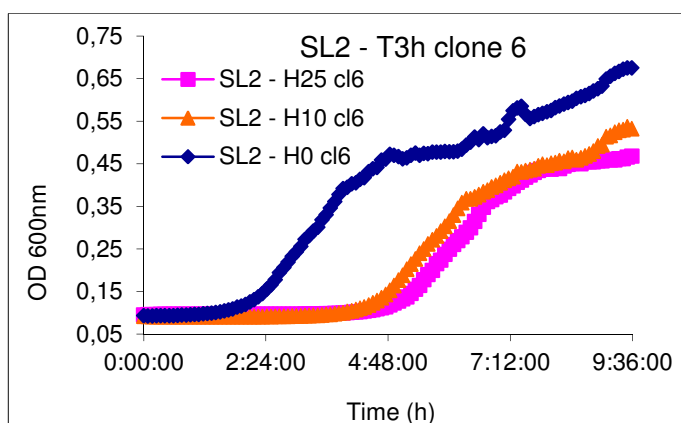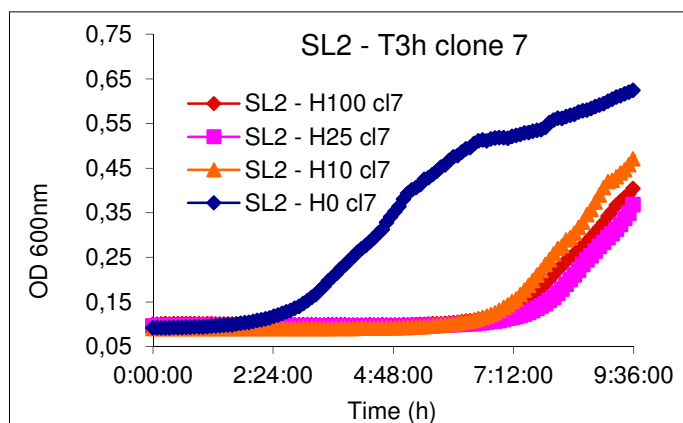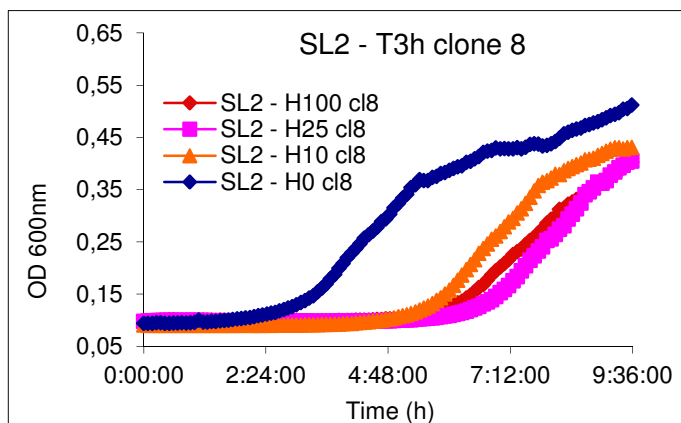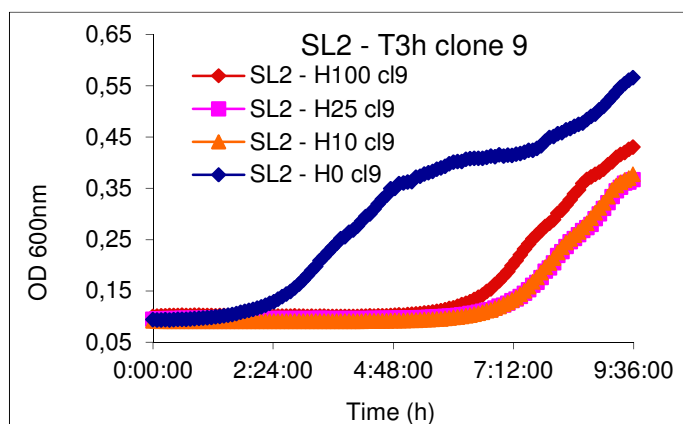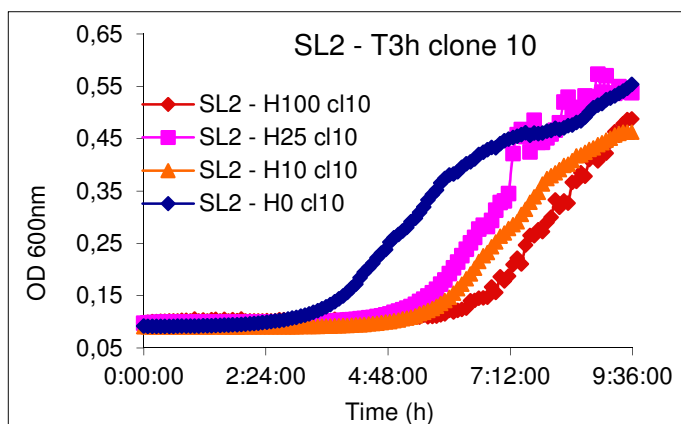

Supplement: Figure S1 — Growth curves of E. coli clones pre-incubated with 100, 25, 10 and 0 µg/ml final of calf thymus histones (annotated H100, H25, H10 and H0 and represented by a pink, red, orange and blue curves, respectively). Single clones isolated during histone selection were tested for their histone resistance or susceptibility. As controls, we tested 10 E. coli clones isolated from the non-selected libraries and E. coli MACH1 pSMART-LCKm (empty vector). The bacterial suspensions were adjusted to final concentration of 106 bacteria/ml in HAH medium (HBSS-, casa-amino acid 0.9% and HEPES 10 mM) supplemented with Km 50 µg/ml. The clones were incubated with 100, 25, 10 and 0 µg/ml final of calf thymus histones 1 h at 37°C with agitation. Then, the bacterial growth was allowed by adding 1 volume of 2X LB, CaCl2 10 mM and Km 50 (37°C with agitation). The growth was followed in 96 well-plate by reading the absorbance at 600 nm during 10 h with a reading every 5 min. The growth curves would be dependent of the amount of living bacteria at the end of the incubation with histone, as shown by the profiles of the MACH1 pSMART-LCKm and the isolated clones from the non-selected libraries. The 10 clones isolated from the selected libraries showed higher resistance. SL2+ corresponds to the 1 to 10 clones isolated from the histone-selected libraries. SL2- corresponds to the 1 to 10 clones isolated from the non-selected libraries. MACH1 pSMART-LCKm is the control of E. coli harboring the empty vector. (PDF) [file pone.0071097.s001.pdf]
